# Glaucoma: Biological Trabecular and Neuroretinal Pathology with Perspectives of Therapy Innovation and Preventive Diagnosis

**DOI:** 10.3389/fnins.2017.00494

**Published:** 2017-09-05

**Authors:** Raffaele Nuzzi, Federico Tridico

**Affiliations:** Eye Clinic Section, Department of Surgical Sciences, University of Turin, Ophthalmic Hospital Turin, Italy

**Keywords:** glaucoma, neuroregeneration, neuroprotection, cell-therapy, gene-therapy, laser-therapy, diagnosis, rehabilitation

## Abstract

Glaucoma is a common degenerative disease affecting *retinal ganglion cells* (RGC) and optic nerve axons, with progressive and chronic course. It is one of the most important reasons of social blindness in industrialized countries. Glaucoma can lead to the development of irreversible visual field loss, if not treated. Diagnosis may be difficult due to lack of symptoms in early stages of disease. In many cases, when patients arrive at clinical evaluation, a severe neuronal damage may have already occurred. In recent years, newer perspective in glaucoma treatment have emerged. The current research is focusing on finding newer drugs and associations or better delivery systems in order to improve the pharmacological treatment and patient compliance. Moreover, the application of various stem cell types with restorative and neuroprotective intent may be found appealing (intravitreal autologous cellular therapy). Advances are made also in terms of parasurgical treatment, characterized by various laser types and techniques. Moreover, recent research has led to the development of central and peripheral retinal rehabilitation (featuring residing cells reactivation and replacement of defective elements), as well as innovations in diagnosis through more specific and refined methods and inexpensive tests.

## Introduction

**Figure 1:**
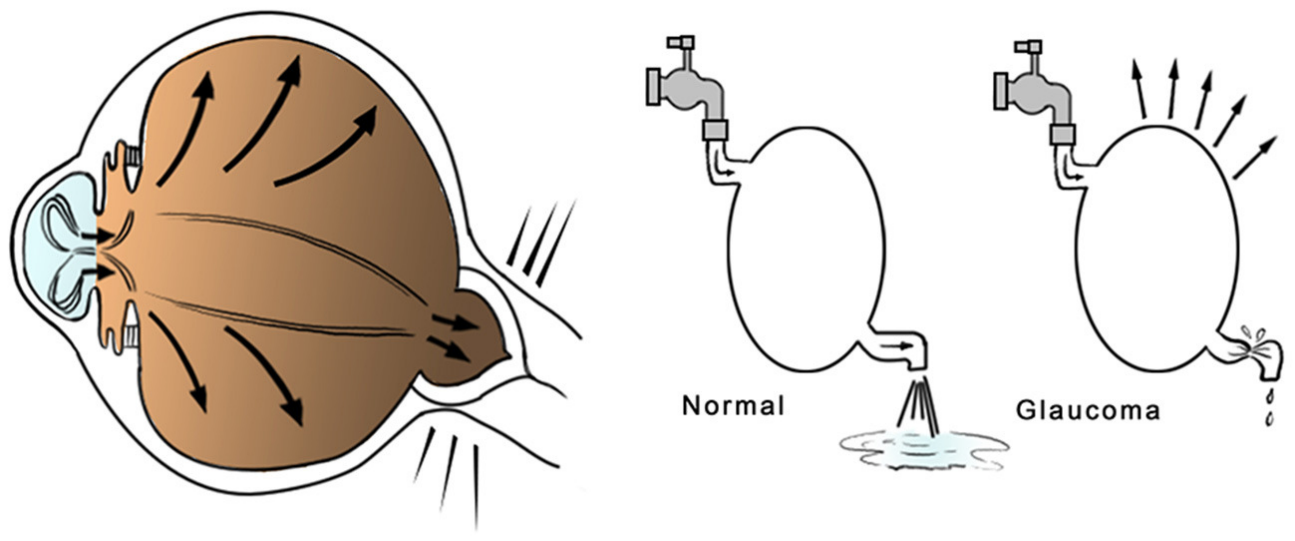
Glaucoma pathophysiology. The glaucoma is a progressive disease related, in most cases, to intraocular pressure (IOP) elevation, affecting the optic nerve and its retinal fibers and causing a progressive loss of vision if untreated. Hyperproduction or low aqueous humor deflow may lead to severe damage to the optic nerve head and optic nerve fibers.

Glaucoma is a common degenerative disease affecting the *retinal ganglion cells* (RGC) and the optic nerve axons, with progressive and chronic course. It is one of the most important reasons of blindness in industrialized countries. Glaucoma can lead to the development of irreversible visual field loss, if not treated (Quigley and Broman, 2006). Diagnosis may be difficult due the lack of symptoms in early stages of disease. In many cases, when a patient arrives at clinical evaluation, a severe neuronal damage may have already occurred. Several studies have calculated that more than half of patients with glaucoma isn't aware of being affected. (Whitson, 2007). Pathogenesis and risk factors of glaucoma are multifactorial: the most relevant risk factor is represented by elevated *intraocular pressure* (IOP) (Figure 1), but familiarity, genetic patterns, race, age, and cardiovascular diseases play an important role, too (Coleman and Miglior, 2008).

Traditional treatment is based on IOP reduction through several methods. The first line approach is pharmacological. Drugs currently in use belong to five different classes and are available in oral and local forms. There are several problems related to this choice of treatment, especially those regarding the low tolerability to some active ingredients and patience compliance. In case of resistance to the medical therapy parasurgical laser treatment is also available: YAG-laser iridotomy/iridectomy or Argon-laser trabeculopasty/gonioplasty. These procedures have the aim to mechanically increase the aqueous humor outflow with preventive or therapeutic intent. Although non-invasive and well tolerated, the efficacy of laser trabeculoplasty may decrease over the years with the need of treatment repetition/extension. Moreover, this procedure is sometimes associated with early IOP spikes, ocular inflammation, iridocorneal synechiae and trabecular scarring. The next step is represented by surgery, based on procedures like ab-externo trabeculectomy and valve implants. To date, these techniques provide a good level of safety and tolerability, but are invasive and not without complications that can be invalidating in some cases (King et al., 2013). As last resort, destructive maneuvers are possible, such as laser photocoagulation, cryotreatment or thermocoagulation of ciliary corps for eyes with uncompensated glaucoma, unresponsive to any treatment (Gupta, 2008).

In recent years, newer perspectives in glaucoma treatment have emerged. Regarding pharmacological treatment, the current research is focusing on the development of innovative mechanisms and/or the improvement of drug efficacy and tolerability, in order to achieve better patients' compliance. For this purpose, current objectives are the improvement of existing therapy, the design of newer drug associations and the development of innovative drug delivery systems, as well as the study of alternative substances (for example drugs with neuroprotective effects).

Great interest in the last years has been dedicated to the treatment of glaucomatous optic neuropathy, with great regards to the clinical and biological research for cell therapy. Their possible application is studied at different levels in order to take advantage of the possibility of autologous transplant with both substitutive and protective intent on neuroretinal elements.

Moreover, recent research has led to the development of central and peripheral retinal rehabilitation, as well as innovations in diagnosis through more specific and more detailed methods. For example, abnormal pupillary light responses can reveal early retinal dysfunction, and it has been observed that blue-yellow dyschromatopsia is prevalent particularly in patients with primary open-angle glaucoma. Therefore, additional diagnostic information may derive from deep investigation of the relationship between glaucoma, lighting and color vision (Nuzzi et al., 1997). Trends for glaucoma treatment and preventive diagnosis covered in this review are summarized in Table 1.

**Table 1:** Summary of glaucoma biological treatment, rehabilitation and diagnosis trends covered in this review.

**MEDICAL TREATMENT**	
-Existing drugs improvement	
-Newer associations	
-Novel drug delivery systems	
	* -Ocular inserts*
	* -Surgical implants*
	* -Soft medicated contact lens*
	* -Nanospheres*
-Innovative hypotensive drugs	
	* -Latrunculinic derivates*
	* -ROCK inhibitors*
	* -BkCa ionic channel modulators*
	* -A1 receptors agonists*
	* -Cannabinoids*
	* -Local calcium channel blockers*
	* -SiRNAs*
-Antioxidative agents	
	* -Ginko biloba extracts*
	* -Resveratrol*
	* -Alpha-lipoic acid*
	* -alpha-luminol*
	* -Stanniocalcine-1*
-Neuroprotective agents	
	* -Memantine*
	* -Brimonidine*
-Neurotrophic growth factors	
	* -Intravitreal GFs (CNTF, BDNF, NGF, GDNF)*
	* -Topical NGF*
	* -Slow releasing implants*
	* -Gene therapy (viral/non-viral vectors)*
	* -Peptidomimetic ligands of TrKA*
**CELL THERAPY**	
-Retinal cell replacement	
	* -Embryonic stem cells*
	* -IPSCs*
	* -Adult stem cells (neural stem cells, retinal precursor cells, ciliary epithelium stem cells, trabecular inserts, MSCs)*
-Neuroprotection	
	* -Intravitreal MSCs*
	* -Encapsulated stem cells*
**OPTIC NERVE AXONAL REGENERATION**	
-Peripheral nerve graft	
-Cell enriched scaffolds	
-Neural growth factors	
	* -BDNF*
	* -CNTF*
-Intracellular signaling	
	* -cAMP induced macrophage activation*
	* -Toll-like receptor 2 agonists*
	* -ROCK inhibitors*
	* -Alpha-crystallins*
	* -Gene therapy*
**PARASURGICAL LASER TREATMENT**	
-Femtosecond pulsed laser	
-Selective laser trabeculoplasty	
-Diode/micropulsed diode laser trabeculoplasty	
-ab interno laser trabeculectomy	
**VISUAL REHABILITATION**	
-Electric artificial stimulation	
	* -Epi-retinal implants*
	* -Sub-retinal implants*
	* -Trans-choroidal implants*
	* -Optic nerve implants*
	* -Cortical implants*
**DIAGNOSIS REFINEMENT**	
-Optical coherence tomography/angiography OCT	
-DARC	
-Telemetric contact lenses	
-Genetic risk assessment	

## Medical treatment

### Current glaucoma treatment

Pharmacological treatment of glaucoma lowers IOP by reducing aqueous humor production and/or improving its deflow. Five pharmacological classes are currently utilized in the treatment of this disease: beta-blockers, prostanoid analogs, alpha-agonists, carbonic-anhydrase inhibitors and cholinergic agents. Initial treatment usually requires a beta-blocker or a prostanoid analog, second-step therapy is based on alpha-agonists and carbonic-anhydrase inhibitors; the last resort is based on cholinergic miotic drugs (Lee and Higginbotham, 2005; Conlon et al., 2017).

In case of not complete IOP control after several tries with different molecules, guidelines suggest the use of associations between different agents with complementary actions. The concurrent administration of systemic carbonic anhydrase inhibitors is also possible.

Problems related to the medical treatment for glaucoma are numerous and cannot be overlooked. Moreover, patients usually show little compliance to treatment, because they often underestimate the situation and do not tolerate multiple instillations of eye drops per day, for an asymptomatic pathology. Moreover, many patients are elderly and unable to practice an efficient administration.

Furthermore, chronic usage of these drugs is related with ocular surface discomfort and modifications, due to the preservatives in pharmacological preparations. Some substances, such as prostanoids have pro-inflammatory effects and may lead to ocular irritation or other annoying or invalidating adverse effects such alterations of iris, eyelids or eyebrows (Stewart et al., 2011).

Conjunctival specimens from patients with collapse of the filtering bleb following filtration surgery show epithelial metaplasia, connectival fibrosis and chronic subclinical inflammation of the conjunctiva, often associated with aberrant expression of some antigens on fibroblasts, macrophages and Langerhans cells. These histological changes may be found even in short-term medicated patients, suggesting that other factors in addition to medical treatment are involved in failure of filtration surgery. On the basis of these immunohistochemical results, we hypothesize that an individual predisposition to abnormal scarring may be involved. The first factor that determine the development of this condition is antiglaucoma long –term topical medication. These alterations could be detected by analysis of preoperative conjunctival biopsy (Nuzzi et al., 1993, 1994, 1995; Nuzzi and Finazzo, 1996a,b; Vercelli et al., 1996). Current research is focusing on the development of innovative mechanisms as well as tolerance and therapeutic efficacy improvement, in order to achieve a better compliance.

### Existing drugs improvement, newer associations and delivery systems

Current research objectives include an efficacy improvement in lowering IOP through innovative systems and/or a greater tolerance, with consequent compliance improvement. In particular, the research is focusing on perfecting existing drugs, through creation of newer associations and development of innovative delivery systems. For example, a partial agonist of prostaglandin A, with different activity in different ocular tissues, may minimize prostanoids effects on ocular surface vessels, lowering conjunctival redness (Hoyer and Boddeke, 1993; Woodward and Chen, 2007).

Newer associations between prostaglandin analogs and carbonic-anhydrase inhibitors are currently in developing stages. Also, other “second generation” therapies capable of lowering local and systemic adverse effects are being developed (e.g., ocular-selective beta-blockers and prostaglandin analogs with alternative and better tolerated preservatives or preservative-free formulations) (Fogagnolo and Rossetti, 2011; Lee and Goldberg, 2011).

Patient adhesion to treatment with eye drops may be often limited, reducing its efficacy. Several delivery systems have been developed in order to improve treatment compliance and efficacy as well. Ocular inserts are designed to administer drugs for several days. One among the best known and studied inserts is the Ocusert system, formed by two polyethylenecovinylacetate membranes and a pilocarpine-filled ring, meant for being applied in the inferior conjunctival fornix, and capable to release the drug within 7 days (Macoul and Pavan-Langston, 1975). Although the efficacy of this system, several patients referred loss of the device or ocular discomfort. This inconvenience led to an improvement of the original design (Pollack et al., 1976; Saettone and Salminen, 1995). Bimatoprost-loaded chitosan inserts have been produced, showing a sustained IOP lowering *in vivo* in rats (Franca et al., 2014). Ocular inserts may be designed for delivering other molecules, such as timolole, but they require patient training to their correct use, limiting their application to younger patients (Stewart and Novak, 1978; Urtti et al., 1994).

Surgical implants (similar to currently used intravitreal implants) may be able to release drugs for longer periods (3–6 months), however their introduction (or removal in case of adverse effects) require invasive surgical procedures, and for this reason their use is more desirable for neuroprotective purposes than as an alternative to existing topical treatment (Lavik et al., 2011). Recently, an intracameral implant for sustained release of bimatoprost has been developed, showing favorable efficacy and safety in a phase II clinical trial (Lewis et al., 2017). Other future perspectives are represented by the development of more sophisticated surgical implants that can be administered with mini-invasive techniques even in ambulatory regimen and that can last for 3–4 months, with the possibility of replacement at the time of follow-up examinations (for example microelectromechanic sub-conjunctival implants, easy to reload and adjust), but long-term studies are needed in order to evaluate function and risks (Staples et al., 2006; Saati et al., 2010).

Regarding soft contact lens usage as a delivery system, the most relevant limitation is represented by the necessity of non-stop application for long periods of time, in order to obtain effective results. Moreover, hydrophilic molecules, such as anti-glaucoma drugs, tend to reflow from highly hydrated polymers of the lens (Peppas et al., 2000). However, contact lenses with N,N-diethylacrilamide, metacrilic acid or acrylate hydrogel polymers have shown a prolonged delivery of timolole and a greater IOP reduction (Hiratani and Alvarez-Lorenzo, 2002; Maulvi et al., 2016).

Other innovative delivery systems are represented by liposome carriers or nanospheres (which provide a greater drug distribution time in corneal tissues, but do not eliminate the fundamental problem of patient compliance when administered through eye drops) and slow-release formulations administered via sub-conjunctival or intracameral injections (Monem et al., 2000; De Campos et al., 2003; Mansoor et al., 2009; Lee et al., 2017). The application of microspheres has been investigated for ameliorating L-dopa induced dyskinesia in Parkinson's disease, with promising results in rat models (Yang et al., 2012; Xie et al., 2014). These drugs provide a longer release, especially if associated with polyester polymers and microspheres (Mansoor et al., 2009; Cardillo et al., 2010). Since erythropoietin (EPO) possesses neuroprotective effects against central nervous systems lesions (Signore et al., 2006; Qi et al., 2014), EPO-loaded microspheres have been tested (both *in vitro* and *in vivo*) on RGCs of rats. In fact, EPO is capable of stimulating neural growth in rat retina explants through EPO-receptors on RGC (Böcker-Meffert et al., 2002). It was observed an improvement on murine RGCs survival after intravitreal and intraperitone administration of EPO-loaded microspheres, which provided sustained neuroprotection, in relation to prolonged release of EPO at the retinal level (Rong et al., 2011, 2012). The principal drawbacks of this technique are represented by local immune reactions of non-degradable polymers (such polyethylene-covinyl-acetate) and lesser efficacy and inconstant drug delivery of degradable polymers of polylactacte (Okabe et al., 2003; Bao et al., 2006).

To date, the clinical efficacy of newer delivery systems is limited by their low effects in terms of bioavailability, compliance and frequent adverse effects. Furthermore, the application of more refined system requires further studies.

### Innovative hypotensive drugs

With the grater comprehension of processes involved in aqueous humor production, innovative ocular hypotensive drugs, with specific molecular targets, have been developed and are currently under evaluation in several clinical trials.

Latrunculinic derivates—macrolides that can inhibit actine polymerization—have provided a greater trabecular meshwork activity through actine cytoskeleton disruption, in studies on animal models and post-mortem analysis, after topic or intracameral administration (Peterson et al., 2000; Okka et al., 2004). Latrunculin B has been evaluated in a phase I study, showing a significant IOP reduction in treated eyes (Rasmussen et al., 2014). However, these molecules are affected by little efficacy and solubility. It has been suggested that their effects can be potentiated by adopting different delivery systems (Chen J. et al., 2011).

Several molecules belonging to the class of RHO-kinase associated protein inhibitors (ROCK inhibitors) have been evaluated in clinical trials (Zhang et al., 2012). ROCK is an effector of the RHO-dependent transduction pathway. Transmembrane receptors and their ligands (growth factors and lysophosphatidic acid) activate RHO-GTPase which activates ROCK. Then ROCK stimulates myosin light chain (MLC) phosphorylation which induces cytoskeletal changes, cell motility, and smooth muscle contraction. Trabecular meshwork cells possess smooth muscle-like properties, as evidenced by the expression of a-smooth muscle actin (a-SMA) (de Kater et al., 1990), and their contraction/relaxation status has been reported to influence aqueous humor outflow facility (Wiederholt et al., 2000). Interestingly, smooth muscle cell contraction is regulated predominately by the phosphorylation status of MLC, a main downstream target of ROCK. Inhibitors of ROCK and Rho GTPases interferes with these processes, reducing IOP in animal models (Collaborative Normal-Tension Glaucoma Study Group, 1998; Tokushige et al., 2007; Van de Velde et al., 2014). Moreover, several studies on animal models have shown that ROCK inhibitors may provide beneficial effects in terms of prevention of scarring tissue formation after filtration surgery, neuroprotection, axonal regeneration and regulation of ocular blood-flow (Van de Velde et al., 2015). Due to their important effects on blood pressure, recent developments of ROCK inhibitor drugs have been limited to topical applications. However, even when applied topically to the eye, side effects, such as conjunctival hyperemia are observed (Tokushige et al., 2007; Tanihara et al., 2008; Mandell et al., 2011) probably due to low specificity or interference with off target ROCK-dependent cellular processes (Tanihara et al., 2008; Williams et al., 2011). “Soft” ROCK inhibitors (locally acting drugs designed to be stable in the desirable site of action and to undergo metabolic inactivation by conversion into a nonfunctional metabolite) have been developed to overcome these issues (Bodor and Buchwald, 2008). As a result, off target activity is avoided resulting in a better safety profile (Boland et al., 2013). It is still uncertain whether the effectiveness of these agents can overcome their adverse effects and therefore their use seems to be limited by the onset of conjunctival hyperemia and subconjunctival hemorrhage. Other potential effects of such drugs are represented by neuroprotection from N-methyl-D-aspartate (NMDA)-induced toxicity, improved survival of ganglion cells and axonal regeneration, as well as an increase in ocular blood flow and inhibition of tenonian fibroblast proliferation (Kitaoka et al., 2004; Honjo et al., 2007).

Another possibility of pharmacological approach is represented by enhancing the levels of nitrogen monoxide (NO), whose release in trabecular environment activates ion channels (particularly a calcium-dependent channel for the potassium, BKCa, that is supposed to alter the conformation of cytoskeleton proteins, myosin and tubulin, in addition to provoke relaxation of smooth muscle cells). However, since excessive NO release may lead to the formation of peroxynitrite, thus increasing oxidative stress, direct stimulation of these ion channels through alternative ligands may provide a more desirable solution (Wiederholt et al., 1994; Siu et al., 2006). Examples of drugs active on this system are combined agonists of PGF2a/NO (prostaglandin analogs capable of releasing NO, obtaining a reduction of IOP greater than simple analogs of prostaglandin) and the ionic channels modulator DNB-001 (currently under evaluation in phase III studies) (Bosworth et al., 2009; Gabelt et al., 2009; Weinreb et al., 2015, 2016; Medeiros et al., 2016).

Other drugs that increase the elimination of aqueous humor in animal and human models are adenosine A1 receptors agonists (Zhong et al., 2013). Adenosine is involved in cellular signaling in stress periods (such as retinal ischemia and elevated levels of IOP) and, by binding to the A1 receptor, promotes the secretion of matrix metallopeptidase-2 (MMP-2), resulting in phospholipase C and G-proteins activation which increase the trabecular meshwork activity (Shearer and Crosson, 2002; Husain et al., 2007).

There is a large amount of experimental data showing the IOP reduction properties of cannabinoids (endo-cannabinoids, synthetic cannabinoid or those derived from plants). The cannabinoid receptor 1 (CB1) was detected in the trabecular meshwork and ciliated epithelium, supporting the role of their agonists in reducing IOP (Pate et al., 1998; Song and Slowey, 2000; Cairns et al., 2016). Cannabinoids may have a direct effect on ciliary processes, dilating blood vessels. This phenomenon could alter aqueous humor dynamics. These molecules are also able to induce cyclooxygenase-2 (COX-2) and prostaglandin E2 expression and consequently the expression of MMP-1, -3, and -9, involved in the aqueous humor deflow (Rosch et al., 2006).

Local administration of calcium channel blockers, such as verapamil, was associated with ocular outflow enhancement in animal models and humans (probably mediated by the block of L-type and T-type calcium-dependent channels) but their use is limited by systemic effects including severe bradycardia and blood hypotension (Erickson et al., 1995).

Other molecules are currently under development or preclinical evaluation for their potential effects in lowering IOP by increasing trabecular outflow or delaying its production. Examples of these drugs are represented by angiotensin II receptor antagonists, 5- hydroxytryptamine receptor 2 (5-HT2) agonists, beta-adrenergic receptor small interfering RNAs (siRNAs), anecortave acetate (a steroid agonist that seems to counter ocular hypertension by inhibiting plasminogen activator inhibitor-1, although his precise mechanism of action is still unclear) (Lee and Goldberg, 2011; Ruz et al., 2011; Zhang et al., 2012). RNA interference regulates the gene expression by modulating protein synthesis with posttranscriptional gene-silencing mechanism. The siRNA enters the cell cytoplasm, then it is incorporated into a protein complex which binds the target RNA messenger inducing its repression and/or cleavage (Fire et al., 1998). Progress with siRNA has been achieved in the field of neurodegenerative conditions, such as Parkinson's and Alzheimer's diseases (Chen et al., 2013; Ma et al., 2013) and the eye is considered a suitable target because it is a confined compartment and, enables local siRNA delivery by topical administration or intraocular injections. Novel molecular strategies protecting siRNAs from degradation and suitable for long-term delivery would open up new perspectives in the treatment of eye diseases, for example retinitis pigmentosa, age-related and diabetic neovascular retinopathies and glaucoma (Guzman-Aranguez et al., 2013). A phase II clinical trial evaluating the effects of SYL040012 (bamosiran, a topically instilled siRNA targeting β-adrenergic receptors) has been concluded on January 2016 (NCT02250612). Preliminary results showed that the treatment is safe and efficient in lowering the IOP, especially in subjects with greater baseline values (Moreno-Montañés et al., 2014).

New perspectives for future drug development to counter ocular hypertension by modulating aqueous humor dynamics derive from the identification of other specific targets, including the melatonin receptor 3 (MT3) (whose action would reduce IOP in primates and rabbit models), endothelin-1 (powerful endothelial vasoconstrictor that antagonizes the effects of NO) and the P2X2 receptor (subtype 2 of the 2X purinoceptors family, a ionotropic channel for nucleotides, promoter of a cholinergic response that stimulates trabecular smooth muscle release) (Lee and Goldberg, 2011).

### Excitotoxicity, oxidative stress, mitochondrial dysfunction, and neuroprotection

In recent years, an important focus on oxidative stress and mitochondrial dysfunction as a cause of glaucomatous neurodegeneration has been carried out. It is assumed that the concentration of free oxygen radicals and other cell death mediators (Tumor Necrosis Factor-alpha, inflammatory cytokines, etc.) increases during inflammatory responses as a result of ischemic insults (prolonged and transient as well) or blood-ocular barrier microalterations, occurring especially under stressful events, such as ocular hypertension (Vohra et al., 2013).

Reactive oxygen species (ROS) can act directly, causing retinal cells death, as well as indirectly as mediators, second messengers or by modulating the activity of other proteins (Izzotti et al., 2006; Tezel, 2006; Chrysostomou et al., 2013). Moreover, oxidative stress contributes to the damage of astrocytes and Müller cells, resulting in excessive glutamate response in the neural synapsis with consequent NMDA hyperactivity disorder related to calcium-dependent apoptotic signaling and dysregulation of metabolic processes that may lead to RGC cytotoxicity (Adachi et al., 1998; Vohra et al., 2013). Dysfunction of glial cells during glaucoma stimulates the production of additional cell death mediators, such as TNF-alpha, and promotes NO increase (Tezel and Wax, 2000).

Another probable cause of increased oxidative status during ischemia is related to mitochondrial dysfunction. In fact, damage to mitochondrial DNA increases with age and reduced ATP production in RGC, compromising their viability. Reduced mitochondrial energy metabolism, promoted by alterations of electron transport cascade, stimulates superoxide and other free radicals' synthesis. High concentrations of these molecules may lead to oxidative damage of macromolecules (such as DNA, proteins and lipids) thus resulting, in conjunction with energy deficiency and dysregulation of intracellular calcium, in neuronal degeneration (Tezel et al., 2009). It is also thought that mitochondrial dysfunction may lead to neuronal death due to production of apoptotic cell mediators through triggering of caspase-dependent processes (Tezel and Yang, 2004).

Thus, the limitation of oxidative stress could be an effective mean in order to obtain a form of neuroprotection and reduce ischemia-related damage. Antioxidant/antiapoptotic agents as alpha-luminol, Ginkgo biloba extracts, resveratrol, stanniocalcine-1 and alpha-lipoic acid have been evaluated in mouse models, proving to be effective in RGC protection (Hirooka et al., 2004; Gionfriddo et al., 2009; Luna et al., 2009; Inman et al., 2013; Kim et al., 2013; Pirhan et al., 2016).

Molecules that have been evaluated in human subjects with neuroprotective intent in glaucoma are memantine (receptor antagonist for NMDA glutamatergic) and brimonidine (an alpha2-adrenergic agonist). Memantine, by blocking the exocytotoxic process mediated by glutamate, has proven useful in preventing the loss of RGC in animal models (Hare and Wheeler, 2009; Ju et al., 2009). To date, two randomized Phase III clinical trials designed to evaluate the efficacy of memantine in reducing the progression of glaucoma have been conducted by Allergan, Inc. (NCT00141882 and NCT00168350, 2009). The results of the first study have not been published, but two subsequent reviews have reported satisfactory outcomes (Cheung et al., 2008; McKinnon et al., 2008). Reports of the second trial showed a significantly lower progression of disease in patients treated with high doses of memantine, compared to those treated with low doses, but there were no significant differences compared to the group receiving placebo (Sena and Lindsley, 2017).

The potential neuroprotective mechanisms of brimonidine include increased activity of brain-derived neurotrophic factor (BDNF) and ciliary neurotrophic factor (CNTF), activation of cell survival pathways (such as reduced expression of mitochondrial transcription factor A, involved in cellular oxidative phosphorylation) and antiapoptotic genes, inhibition of glutamate release induced by ischemia and prevention of oxidative damage caused by exocytotoxic response mediated by NMDA receptor (Wen et al., 1996; Gao et al., 2002; Dong et al., 2008; Lee et al., 2012). In the Low Tension Glaucoma Treatment Study, patients treated with brimonidine showed a lower progression of visual field loss than patients receiving timolol (Krupin et al., 2011). However, a recent Cochrane review has found that the study results were not decisive, given the vast amount of missing data in the group treated with brimonidine (Sena and Lindsley, 2017).

New pharmacological approaches in neuroprotection for glaucoma and other optic neuropathies are currently in development (such as siRNA inhibiting caspases cascade, NO-synthase inhibitors, drugs and synthetic polypeptides with immunomodulatory function) (Neufeld et al., 1997, 2002; Neufeld, 2004; Lee and Goldberg, 2011). Further studies are needed in order to determine with greater certainty whether neuroprotective agents can bring benefits in terms of cell survival/progression of disease in patients with glaucoma.

### Neurotrophic growth factors

Neurotrophic factors, including CNTF, BDNF, neuronal growth factor (NGF), and the glial cell line-derived neurotrophic factor (GDNF) are produced by cells within the retina, however their concentration and the expression of their respective receptors are influenced in complex ways by axonal damage of the optic nerve, increased IOP and introduction of exogenous neurotrophic factors (Perez and Caminos, 1995; Gao et al., 1997; Ju et al., 1999, 2000; Pease et al., 2000; Vecino et al., 2002; Wordinger et al., 2003; Rudzinski et al., 2004). In fact, intrinsic growth factors do not seem to be sufficient in maintaining the viability of RGC in conditions of chronic disease, but exogenous neurotrophic factors may be administered in several different modes.

For example, intravitreal injections of 5 micrograms of BDNF and 2 micrograms of CNTF have reduced the death of RGC in animal models by 8 and 22% respectively after 1 month (Ko et al., 2000, 2001; Ji et al., 2004). As an alternative to repeated intravitreal injections, topical administration of purified neurotrophic factors is possible, but their bioavailability in posterior segment still remains uncertain. However, it was reported that topical administration of NGF four times a day for 7 weeks increases the density of ganglion cells by 37% (Lambiase et al., 2009). It should be noted that this treatment, while showing functional improvements detected with electroretinography, visual evoked potentials and computerized visual field, it was still carried out on a limited number of patients and in the absence of a control group, thus raising doubts about its real efficacy.

Long-term studies on the ganglion cells have unfortunately shown that beneficial effects of neurotrophic factors are temporary, slowing but not preventing cell death. It is supposed that such occurrence is due to receptors down-regulation at the cellular level, thus raising the need for repeated administrations (Harvey et al., 2006; Johnson et al., 2011). The administration of neurotrophic factors can be maintained in time by making advantage of slow release devices implantation. However, while prolonging the time between one treatment and the other, this solution does not seem to eliminate the prospect of repeated applications, as only limited quantities of a particular neurotrophic factor can be released (Jiang et al., 2007; Ward et al., 2007; Johnson et al., 2011). However, administration of biodegradable microspheres may be inconvenient due to the need for several intraocular injections. Therefore, less invasive and painful neuroprotective approaches to support RGCs survival are required.

The gene therapy approach to elevate endogenous retinal production of neurotrophic factors avoids the obstacles associated with the *in vivo* delivery of proteins and peptides and shows promising preclinical results in many retinal neurodegenerative disorders, including glaucoma (Nafissi and Foldvari, 2016). An innovative method by which neurotrophic factors can be given is the use of viral vectors (such as lentivirus, adenovirus, cytomegalovirus and adeno-associated virus) that integrate within the target cells, increasing the endogenous production of neurotrophic factors in the retina (Di Polo et al., 1998; Schmeer et al., 2002; Pease et al., 2009). Adeno-associated vectors (AAV) for gene therapy have already been applied with encouraging results (and relatively rare adverse effects) in children with retinal degenerations due to *Retinal pigment epithelium-specific 65 kDa protein* (retinoid isomerohydrolase, RPE65) mutations (Ku and Pennesi, 2015; Bennett et al., 2016; Weleber et al., 2016) jumpstarting a similar approach for other neurodegenerative ocular diseases. While proving to be effective and well tolerated in animal and experimental glaucoma models, the effects of this method have been transient, probably due to short duration of viral vectors gene expression and triggering of important inflammatory reactions or insertional mutagenesis. New viral vectors with different serotypes, currently under evaluation for other conditions as Leber optical congenital amaurosis, may represent a new twist given their greater efficiency in gene transduction (requiring lower doses and producing longer lasting effects) (Maguire et al., 2008, 2009; Petrs-Silva et al., 2009; Simonelli et al., 2010). Since the capsid protein of AAV is responsible for its tropism toward specific target cells, pseudotyping strategies were developed enabling the packaging of an AAV2 genome into the capsid of another serotype (Rabinowitz et al., 2002). These vectors have the combined advantage of safety and long-term expression of AAV2 and the improved *in vivo* efficacy and tropism of the novel serotypes. The differences in cellular specificity may reflect differences in the expression of viral receptors on the surface of various cell types. After intravitreal injection, only AAV featuring serotypes 2 and 8 emerged as vectors able to efficiently transduce inner retinal layers, while 1, 4, 5, 7, and 8-based vectors delivered transgenes to the neural retina and pigment epithelium after subretinal injection (Auricchio et al., 2001; Lebherz et al., 2008). The vectors able to transduce RGC in rodent models (after both intravitreal and subretinal injection) are those featuring serotype 2 and 8 (Lebherz et al., 2008). The application of vectors specific for RGCs will lead to expanded possibilities for development of ocular gene therapy for glaucoma, due to their cellular tropism.

Newer, more reliable tools (non-viral gene delivery techniques, polysaccharide and liposome nanoparticles, innovative transgenes insertion techniques) avoid the high risks associated with using viral vectors, provide life-long therapy by more policed insertion of the therapeutic gene into the desired site, target a broader range of disorders due to their capability to accommodate genes of different sizes, and finally, provide higher activity owing to their ability to target hard-to-transfect human cells (Nafissi and Foldvari, 2016). For example, Lipopolyplex (a ternary complex of cationic liposome, polycation and DNA) is a novel, non-viral gene delivery vector with high colloidal stability, high gene transfection efficiency and low immunogenicity, capable to cross the blood brain barrier, that has been proposed for neurodegenerative central nervous system conditions (Chen et al., 2016). A similar alternative for gene delivery is represented by the “Trojan Horse Liposome” (THL), an immunoliposome containing DNA and conjugated with monoclonal antibodies, providing high target specificity (Shi et al., 2001). Immunoliposomes loaded with tyrosine hydroxylase expressing plasmids and conjugated with antibodies targeting transferrin receptor ameliorated the striatal tyrosine hydroxylase activity in rat models of Parkinson's disease (Pardridge, 2005). Alternative non-viral vectors may become useful also for gene therapy in glaucoma, since they avoid the adverse effects usually associated with viral vectors, also featuring site and target specificity due to immunological targets or topical administration. Noninvasive topical ocular gene delivery was effectively carried out in a mouse model using eye drops of poly (ethylene oxide)-poly (propylene oxide)-poly (ethylene oxide) (PEOPPO-PEO) polymeric micelles (Liaw et al., 2001).

The use of neurotrophic growth factors for clinical applications was limited also because of their pleiotropic effects (which lead to non-specific cellular responses), toxicity and short half-life. Moreover, neurotrophic factors are distributed inefficiently in target tissues and are not able to pass through the blood-brain barrier. These restrictions can be overcome through the development of peptidomimetic ligands, small molecules, resistant to proteolysis and capable of mimicking binding and activation properties of neurotrophic factors, carrying out their biological activity without the occurrence of side effects. These ligands interact specifically with the appropriate receptor, behaving as agonists or antagonists. Their advantages over native ligands include reduced immunogenicity, low molecular mass, satisfactory pharmacokinetics and high affinity for the target receptor (Johnson et al., 2011).

A peptidomimetic ligand developed to improve the survival of RGC in glaucoma is represented by a TrkA receptor agonist (whose native ligand is NGF which also triggers p57-dependent pro-apoptotic signals) (Shi et al., 2007; Hu et al., 2010). The combination of a selective agonist for the TrkA with an inhibitor of p57 showed major neuronal protection after optic nerve damage (Lebrun-Julien et al., 2009). In addition, the peptidomimetic ligand for TrkA has provided a significant and sustained survival in experimental models of glaucoma (Shi et al., 2007). Therefore, these molecules have a promising therapeutic potential for glaucoma and other neurodegenerative disorders.

## Stem cell therapy

### Stem cells types and glaucoma

In recent years, stem cells have been the subject of great attention as a potential source of cell replacement in diseases that lead to blindness, such as glaucoma. Two unique stem cells properties are their repairing ability, by dividing themselves for an infinite number of times, and their ability to differentiate into many cell types. Advances in stem cell technology provide opportunities to improve our understanding of glaucoma-related biology and offers the possibility of cell-based therapies to restore sight to patients with important vision loss (Chamling et al., 2016). Stem cells can be classified into three main categories, according to their origin: embryonic stem cells, induced pluripotent stem cells and adult stem cells. Embryonic stem cells have many advantages, such as unlimited capacity for self-renewal and pluripotency, however, their clinical use is controversial for ethical, bureaucratic and biological reasons (related to the known risks of tumorigenesis and immunological rejection).

Induced pluripotent stem cells can provide an autologous transplantation approach, but the main safety issues (due to genomic degradation and oncogenesis related to the integration of retroviral or lentiviral vectors in the production process) and the fact that they retain the epigenetic memory of the cell of origin make their application difficult (Stadtfeld et al., 2008; Lin et al., 2009; Zhou et al., 2009; Kim et al., 2010; Polo et al., 2010). Integration capabilities of the *in vitro* differentiated cells have also been tested by subretinal injections in mice (Tucker et al., 2011; Hambright et al., 2012; West et al., 2012). All these studies assessed terminal differentiation and integration of pluripotent cells-derived photoreceptors and, when possible, functionality, although showing variable results.

Adult stem cells (or progenitor cells) lie in different tissues (bone marrow, limbus, etc.) and they maintain high plasticity, although not being pluripotent. The possibility of autologous transplant (thus avoiding the need for subsequent immunosuppression) and minor ethical implications have recently made this cell type very popular (Mimeault et al., 2007; Lodi et al., 2011). Despite the growing understanding of ocular stem cells biology and properties, their clinical application seems still uncertain. To date, the most effective cell therapy is based on the use of ocular limbal stem cells to regenerate the corneal epithelium (Pellegrini et al., 1997).

Taking into account the above disclosure, stem cells can be isolated, induced into differentiation and then transplanted into the retina, where they can overcome the loss of ganglion cells or photoreceptors. Moreover, progenitor stem cells niches suitable for retinal graft were identified.

Neural stem cells, for example, can differentiate into neurons, astrocytes and oligodendrocytes both *in vitro* and *in vivo* models (Gage, 2000), but not in retinal phenotypes, even if their integration into the receiving retina is still possible (Takahashi et al., 1998; Young et al., 2000). On the other hand, stem cells derived from the retina can differentiate into multiple retinal phenotypes, but seem unable to integrate into the receiver's retina, probably for different environmental conditions between under development and mature retina (Chacko et al., 2000). For donor cells to integrate in retinal tissue, specific molecular characteristics are needed and stem cells should be derived from retinae that have not reached complete maturation. Several studies have shown that retinal precursor cells extracted from embryonic retina of animal models have been successfully transplanted into the subretinal space of mice (MacLaren et al., 2006; Klassen et al., 2007; Bartsch et al., 2008; Cho et al., 2012). All these studies have made use of an “*in vivo*” ocular environment to complete the differentiation into mature photoreceptor cells. Despite the promise, the low numbers of integrating cells hinder a real functional recovery in the transplanted eyes, even if some restoration of vision was observed (Pearson et al., 2012).

Human embryonic stem cells can differentiate into retinal phenotypes (especially photoreceptor) and integrate successfully in mice retinas (Lamba et al., 2006). An important discovery was the identification of human ciliary epithelium stem cells, which are able to integrate successfully into the retina and to differentiate into photoreceptors, bipolar, ganglion and Muller cells (opening the possibility for autologous transplant) (Ahmad et al., 2000; Tropepe et al., 2000; Lawrence et al., 2007; Giannelli et al., 2011). Parameswaran et al. (2010) have recently obtained photoreceptors and RGC from fibroblasts induced into pluripotent stem cells.

However, before taking in consideration the application of stem cells to replace RGC, thus repairing the optic nerve damage in glaucoma, additional challenges must be addressed: it is necessary a better understanding of RGC natural differentiation pathways as well as transplanted stem cells stimulation methods that may trigger axonal growth through the damaged optic nerve, carrying out functional synapses with specific cortical targets, thus restoring vision. Furthermore, the heterogenous nature of RGC (Sanes and Masland, 2015; Baden et al., 2016) in terms of physiological, morphological and molecular criteria must be taken into consideration when processing stem cells differentiation protocols in order to replace different types of RGCs.

Another niche of stem cells that could be of great clinical relevance in case of glaucoma is represented by progenitor cells located in the transitional zone between the corneal endothelium and trabecular meshwork, known as the Schwalbe ring (Raviola, 1982; Kelley et al., 2009; Yu et al., 2011). Currently different approaches for the extraction and isolation of these “trabecular inserts” are being evaluated, including surgical dissections with high resolution microscopes and detection of specific cellular markers (Gonzalez et al., 2004; Du et al., 2005; Liton et al., 2005; McGowan et al., 2007). These progenitor cells can replace missing or insufficient trabecular cells in glaucoma patients revealing a potential alternative to prevent the loss of vision and facilitate compliance in place of long term eye drops applicarion or expensive laser treatments. Further researches are required in order to establish a protocol to regulate the division and differentiation of these inserts in the appropriate cell lines.

**Figure 2:**
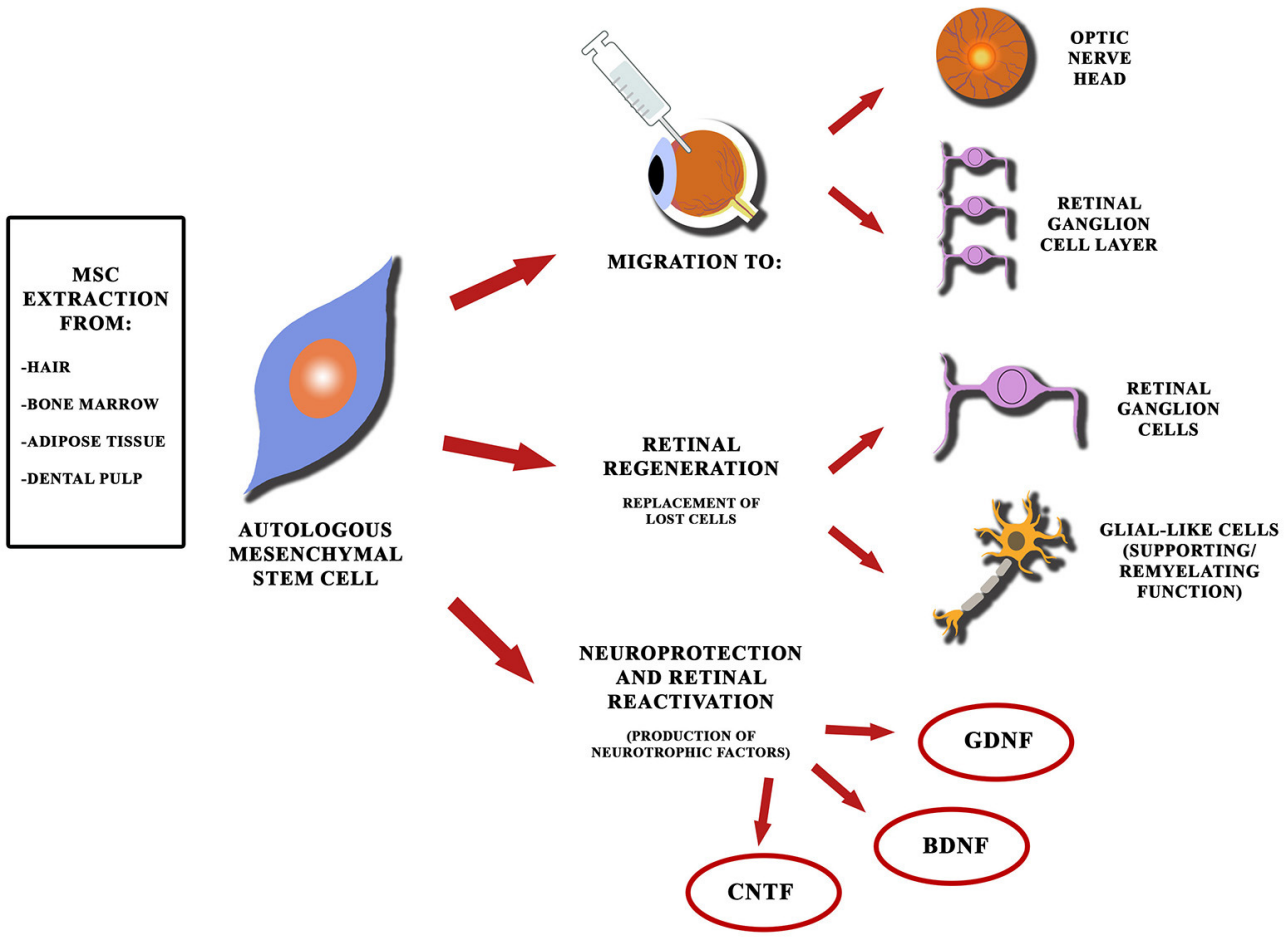
Schematic diagram showing perspectives of mesenchymal stem cells for glaucoma. Autologous mesenchymal stem cells can be extracted from different sources (such as bone marrow and adipose tissue) and can be used to replace retinal cell elements, lost due to glaucomatous injury, since they are able to migrate toward the optic nerve head and the retinal ganglion cell layer (even after intravitreal injection). MSCs can also produce neurotrophic factors providing neuroprotection and reactivation of quiescent cells in the retina.

Autologous mesenchymal stem cells (MSCs) derived from human bone marrow could represent a further source of stem cells for regenerative purposes (Figure 2), given their greater ease of extraction and their ability to migrate to retina and optic nerve head (ONH) after intravitreal injection in murine models. Moreover, it has been observed that MSCs produce neural growth factors after intravitreal injection in animal models (Johnson et al., 2010). As for their use in humans, the experience regarding their application in glaucoma is extremely limited. It's reasonable to suppose beneficial effects also in human glaucoma models, as suggested by Connick et al. (2012), since neuroprotective effects in patients with secondary progressive multiple sclerosis have been observed as a result of intravenous administration of MSC (with improvements in terms of visual acuity, visual evoked potentials latency and the optic nerve area). The Retina Associates of South Florida and MD Stem Cells have developed a clinical trial in order to evaluate the use of autologous stem cells taken from bone marrow in the treatment of various eye diseases, including glaucomatous optic neuropathy (Stem Cell Ophthalmology Treatment Study, NCT01920867). The trial considers comparison of different study groups that will receive administration of MSC through subtenionan, retrobulbar, intravenous and intravitreal ways (with or without associated vitrectomy procedure). Preliminary results have been recently published with encouraging visual acuity improvements in patients affected by optical neuropathies, such as Leber's hereditary optic neuropathy (Weiss et al., 2015, 2016). The conclusion of this clinical trial is estimated for 2017.

### Cell therapy and neuroretinal protection

An alternative use of stem cells is emerging. In fact, recent studies have shown that a large variety of progenitor cells, when transplanted, possess neuroprotective properties in experimental models of glaucoma. It is believed that cell-mediated neuroprotection is conferred by production of various neurotrophic factors. It's evident that oligodendrocyte precursors can reduce by 32% the death of ganglion cells (Bull et al., 2008, 2009) and mesenchymal stem cells can simultaneously produce a wide range of neurotrophic factors (such as BDNF, CNTF, and GDNF), providing a reduction of cell death of 28% after 1 month from glaucoma onset (Li et al., 2002; Ye et al., 2005; Crigler et al., 2006; Yu et al., 2006; Arnhold et al., 2007; Inoue et al., 2007; Li N. et al., 2009; Wilkins et al., 2009; Zwart et al., 2009; Johnson et al., 2010). Other appealing perspectives are possible: to enhance stem cells with genetic engineering techniques in order to produce a significantly greater amount of neurotrophic factors (Kurozumi et al., 2004; Ikeda et al., 2005; Liu et al., 2006; Sasaki et al., 2009).

A recent study by Mead et al. (2014) compared the neuroprotective efficacy of adipose-derived mesenchymal stem cells, bone-marrow derived mesenchymal stem cells and dental pulp mesenchymal cells (DPSC), showing that BMSC and, to a greater extent, DPSC provided significant protection from RGC loss and preserved RGC function in mice after intravitreal injection. The relatively longevity of transplanted intravitreal MSC is likely due to immunosuppressive features of MSC and properties of vitreous body preventing cell migration (Mead and Scheven, 2015; Mead et al., 2015). Even though the results obtained as yet are promising, some important obstacles still need to be overcome, including the rejection of the transplanted cells and potential oncogenesis associated with implantation of undifferentiated cells.

However, both problems can be overcome by the development of semi-permeable capsules that enclose the stem cells, isolating them from the surrounding retinal environment, while maintaining the ability to secrete neurotrophic factors. Ultimately, the interest in encapsulated stem cells has grown even further, leading to the development of clinical trials for diseases, such as retinitis pigmentosa (Sieving et al., 2006) and the dry form of age-related macular degeneration (NCT00447954), and in case of successful result, these studies may suggest a similar approach in glaucoma patients.

### Optic nerve axonal regeneration

In the final stages of glaucoma, the optic nerve is affected by a major atrophy that causes irreversible damage, resulting in irreparable loss of visual function. Optic nerve axonal regeneration after any kind of injury seems to be inhibited by at least three major obstacles: apoptosis of RGC, inability to trigger axonal growth, cellular microenvironment of the central nervous system containing inhibitory factors. Various therapeutic strategies have been evaluated to overcome these obstacles and restore lost functionality, one of which is represented by the transplantation of the optic nerve.

A study of Aguayo et al. (1987) showed that regenerative capacity of mammals' RGC could be facilitated in a more permissive microenvironment obtained with the application of a peripheral nerve graft. The use of a peripheral nerve as a new conduction pathway between the retina and mesencephalic sovratectal nuclei, in association with the application of cellular growth factors, has led to the restoration of the pupillary reflex in mice with extensive lesions of the optic nerve. Negishi et al. (2001) have studied the use of a graft made from a silicone tube enriched with purified Schwann cells, extracellular matrix (Matrigel), NGF and BDNF in murine models subjected to axotomy, observing tissue development and regeneration of blood vessels, RGC and their axons. Other artificial substrates have also been developed with the same purpose. For example, a peptide nanofiber scaffold has been shown to stimulate axonal regeneration with return of functional vision in hamsters (Qin et al., 2013).

**Figure 3:**
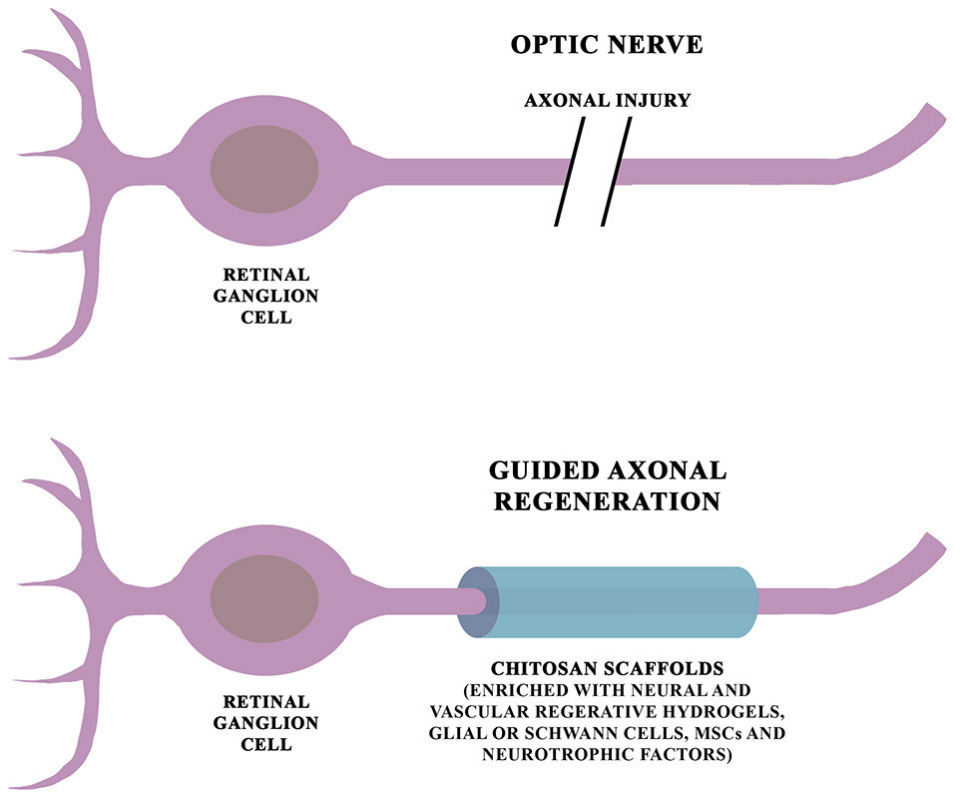
Schematic diagram of chitosan-based scaffolds for glaucoma. Chitosan scaffolds have shown to facilitate axonal repairing and growth in both central and peripheral nervous system. Moreover, their beneficial effects can be promoted by their combination with neural and vascular regenerative hydrogels, adhesion molecules, glial cells, Schwann cells, mesenchymal stem cells and neurotrophic factors.

Various biomaterials have been proposed to obtain scaffolds for promoting axonal repair. Among them, chitosan, a derivate of chitin extracted from shellfishes, has shown biomimetic properties which make it a promising candidate for developing innovative devices for neural repair (Figure 3). *In vivo* experimental studies have shown that chitosan can be successfully used to produce scaffolds that promote neural regeneration in the central and peripheral nervous system (Gnavi et al., 2013; Meyer et al., 2016). Chitosan conduits can also be enriched with adhesion molecules (such as laminin, collagen and L1), mesenchymal stem cells and neurotrophic factors for facilitating nerve regeneration and guiding neurite growth (Cheng et al., 2007; Li X. et al., 2009; Chen X. et al., 2011; Guo et al., 2012). Retinal progenitor cells cultured on cationic chitosan-graft-poly(ε-caprolactone)/polycaprolactone (CS-PCL/PCL) scaffolds shown to differentiate into retinal neurons, suggesting the potential of these biomaterials in retinal tissue engineering (Chen H. et al., 2011). Xu et al. (2004), by using an animal model of optic nerve transection, showed that polyglycolic acid(PGA)-chitosan scaffolds, coated with recombinant L1-Fc, have a potential role in promoting nerve regeneration by guiding axonal regrowth and remyelination. The application of chitosan scaffolds, though promising, must be subjected to further studies to evaluate their use for glaucoma.

Neurotrophic factors, such as BDNF and CNTF enhance survival of axotomized RGC (Mansour-Robaey et al., 1994; Muller et al., 2009) and promote mild axonal sprouting (Shum et al., 2016). Recently, the administration of osteopontin has shown to potentiate significantly the regenerative response of alpha RGC to BDNF stimulation (Duan et al., 2015).

Another way to repair the optic nerve damage as a result of glaucoma consists in triggering neuronal growth by acting on different intercellular signals. Among the interventions tested in experimental models so far, we find macrophage activation induced by c-AMP analogs (a mechanism capable of promoting ganglion cells and axonal fibers proliferation through oncomodulin action and growth factors, such as CNTF), Toll-like receptor-2 agonists (e.g., zymosan that would promote the activation of macrophages, Müller cells and retinal astrocytes involved in the mechanisms of neuronal regrowth), activation of RGC proliferation, as well as optic nerve fibers growth, following lens puncture (a process mediated by the subsequent inflammatory response triggered by the release of lens proteins in the posterior chamber), and ROCK inhibitors (Kurimoto et al., 2010; Fischer and Leibinger, 2012). Neuroregenerative effects of ROCK inhibitors is mediated by the block of ROCK signaling cascade, which is known as a negative regulator of neurite extension and can be activated by several neural growth inhibitors expressed in the central nervous system myelin, such as Nogo-A, MAG and Omgp (Van de Velde et al., 2015). In addition, small interfering RNA targeting the Nogo-66 receptor (NgR, a receptor shared by Nogo-A, MAG and Omgp, triggering the ROCK cascade), in association with an oncomodulin/truncated protamine vector (featuring specific affinity for RGC), has demonstrated axonal regenerating effects on RGCs, in *in vitro* rat models (Cui et al., 2014).

Alpha crystallins are proteins that were first identified as major structural components of the ocular lens, but they also share homology with heat shock proteins and have chaperone-like features, including preventing stress-induced apoptosis (Liu et al., 2004). Several studies reported downregulation of crystallins in various models of glaucoma, suggesting that decreased levels of these proteins may reduce RGC survival (Piri et al., 2007). This hypothesis was corroborated by increased survival of axotomized RGCs in retinas overexpressing alpha A or alpha B crystallins (Ying et al., 2008; Munemasa et al., 2009). In addition to RGC protective functions of alpha crystallins, beta and gamma crystallins were implicated in RGC axonal regeneration (Fischer et al., 2001; Teng and Tang, 2006).

Also, several targets for gene therapy have been studied in order to promote axonal regeneration. In fact, activation of Mst3b, c-myc and RAF-MEK signaling enables and favors neurite growth (Lorber et al., 2009; O'Donovan et al., 2014), while deletion of genes encoding suppressors of neurotrophic pathways, such as CaM kinase, MAP kinase, JAK/STAT, and PI 3-kinase/mTOR have resulted in sustained axonal regeneration (Shum et al., 2016). Moreover, deletion of PTEN and/or SOCS3 genes was found to increase nerve regeneration, allowing regenerating axons to reach optic chiasm from injured optic nerve (Park et al., 2008; Smith et al., 2009; Sun et al., 2011; de Lima et al., 2012).

Future inspiration may lie in fish and salamanders, since they retain the capability for regeneration and neurogenesis after central nervous system injury. Analysis of visual recovery in zebrafish showed two phases of recovery (Kato et al., 2004). In the first phase, retinal projections are limited to the outer layer of the optic tectum, and the fish show a gross optomotor response (Bilotta, 2000). The slow phase allows for recovery of high resolution vision, and involves complete restoration of visual circuits and refinement of synaptic terminals (McDowell et al., 2004). Since many molecular pathways of the central nervous system, are shared, the adult zebrafish is a powerful model to study neural regeneration (Becker and Becker, 2008; Shum et al., 2016). Finally, autologous stem cells, either for regenerative, or neuroprotective intent, could find an interesting application for the same purposes described above, but their effects and their safety profile need to be outlined in greater detail through further studies.

## Parasurgical therapy: the laser treatment

The laser treatment of glaucoma, in its various types, is typically used as second-line therapy, after medical therapy failure or in association with anti-glaucoma drugs. Traditional techniques are YAG laser iridotomy, argon laser trabeculoplasty (ALT) and diode laser cyclophotocoagulation. The first two increase the outflow of aqueous humor by creating filtering spaces obtained by directing destructive laser pulses at the level of peripheral iris or the trabecular meshwork. The last one, however, decreases aqueous humor production by destroying ciliary processes.

The advantages of laser therapy are mainly the low invasiveness, reduced probability of infection, ease of implementation and minimal post-operative pain. Complications are mainly of inflammatory nature, but they also include IOP elevation peaks, aqueous drainage block due to trabecular scarring and corneal damage (Thompson et al., 2002). Research efforts in recent years have been focused on finding new methods for concentrating and directing the laser energy only on interested areas, limiting thermal damage to adjacent tissues.

### Femtosecond pulsed laser

The femtosecond pulsed laser has high intensity and very short duration pulse, making it precisely directable on the target tissue with limited effects on adjacent areas. Ngoi et al. (2005) have studied its application on pig eyes practicing *in vitro* iridotomy: results showed that the power consumption is 90 mW compared to 200 mW of a traditional Argon laser system. The femtosecond laser was studied *in vivo* on rabbit eyes by Chai et al. (2010), with the intent of creating artificial drainage channels at the trabecular and sclero-conjunctival level. In treated eyes, it was demonstrated both the effectiveness on anatomical investigations with optical coherence tomography (OCT) imaging, and functional effectiveness showing a significant reduction in IOP (Chai et al., 2010).

### Selective laser trabeculoplasty (SLT)

In recent years, selective laser trabeculoplasty (SLT) has been proposed as a viable alternative to ALT. It uses a pulsed YAG laser, with very low intensity and selective target on trabecular meshwork pigment cells. Compared to ALT, employed energy and exposure time are lower. Moreover, the selectivity of cellular targets prevents side effects from indiscriminate damage of the trabecular meshwork, preventing scarring with possible formation of synechiae (Realini, 2008). However, the response rates within the first postoperative year have varied from 59 to 96%, according to different definitions and studies. The reported average reduction in IOP from pretreatment IOP ranges from 18 to 40%, over a follow-up period of 6 to 12 months (Jha et al., 2012).

### Diode laser trabeculoplasty (DLT) and micro-pulsed diode laser trabeculoplasty (MDLT)

Another possibility that has emerged in recent years is the diode laser trabeculoplasty (DLT). The advantages of this technology compared to the traditional ALT are a lower energy pulse and greater equipment simplicity and portability. As a matter of fact, ALT uses argon ionized gas, high voltage (350 V), a 20–30 A current and a system consisting of several mirrors, which are prone to rupture or become misaligned. Devices employed in ALT are also very large and only some models are transportable. The DLT instead uses a solid crystal, low-voltage (3 V), a current of 1 to 4 A and a system of mirrors that does not have alignment issues. Dimensions are also very contained and the equipment is fully transportable. Long-term studies have demonstrated similar efficacy in terms of reduction of IOP (Chung et al., 1998). Further research led to the development of a micro-pulsed variant (MDLT) that allows to gain the same results with less side effects (Sivaprasad et al., 2010; Coombs and Radcliffe, 2014). The laser energy, instead of being contained in a single pulse of the duration of 0.1–05 s is split into a series of pulses of 100–300 ms each, for a total exposure time of about 0.1–0.5 s. In this way, the time lapse between a micro pulse and the other allows a partial cooling of the target tissue, to limit overheating and therefore the diffusion of heat and thermal damage to the adjacent areas (Dorin, 2003).

### Ab-interno excimer laser trabeculotomy (ELT)

In recent years, enthusiasm has been aroused by ab-interno excimer laser trabeculotomy (ELT). This technique is based on the creation of micro-perforations that connect the anterior chamber to Schlemm's canal, thus mechanically increasing outflow routes (Vogel and Lauritzen, 1997). It is a minimally invasive technique with rapid execution (4–5 min), which may also be associated to cataract surgery. Compared to trabeculoplasty, it does not induce thermal damage to trabecular structures adjacent to the micro holes and therefore has no complications related to scarring. Studies by Wilmsmeyer et al. (2006) showed a significant reduction in IOP attributable to ELT, especially if employed in association with phacoemulsification procedure.

Laser technologies are constantly evolving, thanks to more advanced level of research, leading to new modalities, such as titanium-sapphire laser and pattern scanning trabeculoplasty (Tsang et al., 2016). Thanks to the practicality of execution and to progressive less incidence of complications, they are spreading not only as a second level treatment, but also as assistance to first-line medical therapy or alternatively as a first choice. Further studies are required to certify their actual efficacy and safety (Meyer and Lawrence, 2012).

## Rehabilitation therapy

Despite the efforts of clinical and biological research, at present time glaucoma, if untreated, leads to blindness or other serious and debilitating impairment of peripheral vision. For these advanced cases, an additional resource is rehabilitation therapy through patient education to the use of residual vision and repeated training through visual stimulation. The rationale of these methods comes from studies regarding the plasticity of the visual system. According to many theories, neuronal damage triggers morphological and functional reorganization processes that would lead to the creation of new neural links or to the use of previously underutilized existing ways. Plasticity is in fact a typical property of nerve tissue, which is expressed not only during the age of development, but also during the whole lifetime, albeit in a more limited fashion. This mechanism is a fundamental process for functional recovery after a damaging event.

An example of what has just been explained is constituted by the phenomenon of blindsight, which is the ability shown by some patients with cortical blindness to respond to visual stimuli presented in the corresponding area of the visual field without perceiving it consciously (Weiskrantz et al., 1974). It is supposed that as a result of visual cortex lesions a spontaneous plastic anatomical reorganization process is put in place. Through the recruitment of subcortical pathways, it is thus possible to balance, albeit weakly, the loss of functionality. In cases, such as the ones shown above, with proper rehabilitation, interventions can consolidate or even improve remaining visual performance, beyond the small, spontaneous recovery that usually occurs in the first few months after acute event.

In addition to the rehabilitation therapy the use of molecules that reduce neuronal death might encourage the process of reorganization and thus increase treatment efficiency. The same plastic/protective processes might be relevant even when the damage affects not only the central point of arrival of the visual stimulus (the cerebral cortex), but also its starting point (the ganglion cells and the optic nerve), as in glaucoma.

To understand more the role of neuronal plasticity in the processes of functional recovery, studies have been conducted using the innovative technique of functional magnetic resonance imaging to assess and confirm the changes that occur in the central nervous system in visually impaired patients after cycles of visual rehabilitation (Nuzzi and Buschini, 2010).

Another line of investigation lies in studying visual rehabilitation by electrical stimulation of different targets: the retinal neuronal cells, the optic nerve or visual areas of the brain (Lorach et al., 2013; Sehic et al., 2016). Not only glaucoma patients are examined in these studies, but also those suffering from retinitis pigmentosa, age-related macular degeneration and diabetic retinopathy, which are among the most frequent causes of blindness, according to the World Health Organization (Pascolini and Mariotti, 2012). However, until now, artificial implants were not able to restore a complete physiological visual function, but they provide a limited and artificial perception. Even so, with its plastic capacities, the nervous system could be adapted to the conduction and interpretation of these new paraphysiological stimuli, with good functional results, even if the technical difficulties and applications are numerous and have not yet completely overcome nowadays.

### Epi-retinal implants

The stimulation targets are the RGC. The electrodes are placed directly on the retinal surface and connected to a device that stimulates the target and at the same time receives the data. The first *in vivo* experiments were conducted between 2003 and 2009 by Humayun and Caspi (Humayun et al., 2003; Caspi et al., 2009), with good results: the subjects were able to recognize the shapes and orientation, with a minimum recovery of visual acuity (20/3,240). Further studies have achieved a better resolution and approval for marketing and distribution in Europe (Humayun et al., 2012). The patients were able to identify orientation, location and movement of objects, with a greater recovery of visual acuity (20/1,260). Despite the efforts, however, all patients remained far below the limit of legal blindness (20/200) and the stimuli were not able to reach areas in order to maintain specific retinotopia. Furthermore, interventions of this type are highly destructive from the anatomical point of view, without possibility of recovery in case of failure.

### Sub-retinal implants

The stimulation targets are the inner nuclear layers. Developed systems have multiple advantages compared to epiretinal ones: they are implantable beneath the retina and therefore more stable; they are completely autonomous because they do not require connection to any type of external device; stimulation thresholds are also lower. Also, multiple independent systems have been studied, in order to increase the coverage of the visual field. Despite all the achievements *in vivo*, there are only slightly improvements in terms of visual acuity (20/1,000, Wilke et al., 2011) with unsolved problems regarding failure to maintain the retinotopia and high invasiveness as well.

### Trans-choroidal implants

In order to bypass the problems of retinal damage, implantation methods below the choroid (Fujikado et al., 2011) or even external to the sclera (Chowdhury et al., 2005) are currently under evaluation. Despite the lesser invasiveness, the disadvantage is represented by greater stimulation intensity required.

### Optic nerve

The stimulation of the optic nerve is an intriguing prospect, since it allows to restrict the target area and reduce the intensity of the stimuli reaching the entire field of vision: in fact, the optic nerve receives information from all areas of the visual field, and therefore it allows to stimulate central and peripheral visual field at the same time. Several implant types have been studied: implants made of 4 or 8 electrodes and placed on the surface around the optic nerve, penetrating electrodes and intraocular implants (Fang et al., 2006; Chai et al., 2008; Brelén et al., 2010; Wu et al., 2010). Various studies have achieved the perception of light stimuli with different spatial orientations, with a high success rate. These devices are less invasive because they are localized in a smaller area, but present greater problems of spatial resolution in comparison with others because of high concentration of fibers in a minimum area. In addition, some devices have shown over time an increase in the stimulation threshold required to evoke stimuli, probably due to development of reactive gliosis phenomena.

### Cortical implants

It is the rehabilitation possibility that is best suited to glaucoma, since in this condition the optic nerve is damaged and therefore no longer perfectly usable as a path of conduction of stimuli.

Dobelle and colleagues studied surface implants placed in the visual cortex managing to achieve a visual acuity of 20/400. The subject under examination was able to recognize large letters, avoid obstacles in the environment, find objects and move in space (Dobelle, 2000). To decrease stimulation thresholds required, penetrative cortical implants were also studied. Studies in primates have given positive results, but with little behavior related response. They are also complicated by high invasiveness of the implant, risk of infection, inflammation, reactive gliosis, and neuronal death (Torab et al., 2011).

An attractive target group is represented by the lateral geniculate body, as it is equipped with high spatial segregation and well known retinotopic organization, divided into the magnocellular and parvocellular pathways. Furthermore, the fovea projects on a larger area, thus precise electrical stimulation would seem easier to be performed. Current studies have shown good results in terms of perception, but are still at an early stage (Pezaris and Reid, 2007).

## Diagnostic refinement

Since functional and anatomic changes due to glaucoma are often irreversible, early detection still remains an important strategy to prevent loss of vision. This goal has been achieved so far evaluating optic nerve structure and function through retinographies and perimetries. New techniques are emerging to complement the use of these consolidated procedures, including analysis of nerve fibers and detection of apoptosis of *in vivo* ganglion cells.

### Optical coherence tomography

The OCT in glaucoma offers the opportunity to objectively measure the optic nerve head, the retinal nerve fiber layer (RNFL) and their changes over time, allowing a fast, non-invasive, highly reproducible and high-resolution evaluation. The OCT ideally allows the detection of morphological changes of the optic nerve head earlier than standard methods. The high reproducibility in RNFL thickness measurement can improve the ability to detect glaucoma in its early stages, by referring small changes (Gonzalez-Garcia et al., 2009; Vizzeri et al., 2009; Shin et al., 2010). Recent advances, with the advent of swept-source OCT, led to improvements in image depth and scan speed, with novel and useful features that can be applied in the field of glaucoma (Lavinsky and Lavinsky, 2016). OCT angiography (OCT-A) is a recent modality that can elaborate a three-dimensional vascularization study exploiting the time-dependent backscattering OCT signal of moving erythrocytes, providing the flow map of blood vessels and capillary plexus in different layers of the retina, without the need of intravenous dye injection (Spaide et al., 2015). OCT-A makes also possible to study capillary plexus of the optic nerve head and adjacent regions, leading to rapid quantification of blood perfusion and opening up new perspectives in understanding pathophysiology of glaucoma (Liu et al., 2015; Bazvand et al., 2017; Na et al., 2017). OCT-A is also able to evaluate early microvascular changes of the ONH in pre-perimetric open angle glaucoma expanding the tools for early diagnosis and follow-up (Cennamo et al., 2017). However, long-term studies are needed to confirm its use in clinical practice.

### Detection of apoptotic retinal cells

Detection of apoptotic retinal cells (DARC) before visual function loss (when approximately 40% of RGC are lost) has been a diagnostic target for several years. A particular technique of DARC makes use of Annexin V (a particular protein that binds phospholipids in the presence of Ca^2+^) to identify “*in vivo*” apoptotic cells using radiologic methods and fluorescence (and therefore without radioactive effects) (Guo and Cordeiro, 2008). This method has shown a good correlation with histological findings in animal models (Tatton et al., 2001; Cordeiro et al., 2004, 2011). For the data collected by the DARC to have actual significance at diagnostic level, studies are needed on samples consisting of patients, in order to match the progression of glaucoma and to assess potential toxicity profiles (although no side effects have been reported in previous clinical trials) (Coxon et al., 2011). In conclusion, DARC can be a promising biomarker for glaucoma diagnosis and follow-up, as well as for analysis of drug therapy effects.

### Telemetric contact lenses

The application of telemetric contact lenses (which are able to detect the IOP fluctuations 24 h a day) may be useful for enhancing the ability to identify patients who require a personalized treatment or which have defects of compliance to therapy. The devices currently in development include a silicone disposable contact lens with a micro-electromechanical system and a built-in titanium micro-caliber which measures changes in corneal curvature (Pajic et al., 2011). This technology is based on the correlation between the changes in IOP and corneal curvature (variations of 1 mmHg in IOP would cause changes in the corneal curvature radius of about 3 microns). The measurements are performed for 30 s every 5 min, with a total of 288 daily surveys (Kersey et al., 2013). Unfortunately, the current device provides results in an arbitrary measurement unit yet to be converted to mmHg (Mansouri and Weinreb, 2012). Other techniques for a continuous measurement of the IOP are under development and although promising clinical trials are needed on a large scale to evaluate tolerability, clinical application and costs/benefits ratio.

### Genetics and prevention

Genetic risk evidence for primary glaucoma came from family linkage-studies implicating a small number of disease genes. Recently, Janssen et al. (2013) reviewed over 120 family and Genome Wide Association studies and selected 65 *primary open-angle glaucoma* (POAG) candidate genes, to assess their role in glaucoma development. It was found that the proteins corresponding to these 65 genes take part in common functional molecular networks related to visual system development, lipid metabolism, connective tissue development and inflammatory processes. Thus, it was shown that taking into account the selected 65 genes substantially increased the specificity and sensitivity of a discriminative primary open angle glaucoma risk test, based on “receiver operator characteristics curves” from the Rotterdam Study I (Ramdas et al., 2010). Since glaucoma follows a polygenic model, the susceptibility to the disease increases with the number of risk alleles that individuals carry in their genome. If we combine this information with environmental risk-factors for glaucoma, an accurate personal risk assessment should be possible and preventive measures or personalized treatment can be applied, based on one's genetic profile. Indeed, further studies are needed to better assess the role of specific genetic backgrounds and their interaction with different pathobiological events that may lead to glaucoma onset.

## The clinical and biological research: final considerations

In recent years, great strides have been made in the research of glaucoma treatment. Newer strategies will get better results with fewer side effects and invasiveness.

The study of alternative pharmacological approaches gives great hopes regarding neuroprotection and cell therapy. To date, however, the practical use is still limited to clinical trials, because coherent results, showing clear efficacy in visual field defects prevention and retinal neuronal cell death decrease, are not available yet. Great efforts have been made regarding animal and cellular research, and the results appear encouraging. Despite this, enormous difficulties remain in terms of practical application, follow-up duration, choice of objectives to be achieved, variability of the disease, patient adherence to treatment and choice of standard methods for measuring the actual treatment effectiveness.

Parasurgical therapies have reached very high levels of accuracy, limiting the destructive effect to desired areas, with minimal involvement of adjacent tissue. Thanks to the ease of implementation and the progressively less incidence of complications, laser treatments that were considered as second choice compared to medical therapy, are now used in addition to it or even in the front line. Moreover, technology is constantly changing, thanks to research at a more advanced level. Further studies are necessary in order to verify objectively the actual efficacy and safety (Meyer and Lawrence, 2012).

Today the rehabilitative therapy is still offered in the final line, for those cases in which no other intervention is found to be effective. Neuronal plasticity is a phenomenon now recognized by all. It is maximum in perinatal age, reduced but still present even in the later stages of life. Recent research is underway to exploit plasticity of residual elements or to unleash and stimulate them in order to achieve some degree of rehabilitation in cases of high pathological impairment. Brand new electronic devices utilize technology capabilities more advanced in terms of efficiency and miniaturization. Despite everything we are still far from widespread use, for the complexity of anatomical structures and the high invasiveness of these devices which create an irreversible disruption of the ocular structures. Nowadays the only assessment tools actually used and tested are the evaluation of the IOP and progression of the visual field defects. The emerging methodologies already in use as the OCT are not very invasive and easily achievable, but long-term studies are needed to confirm its use in clinical practice. Other strategies, such as DARC and telemetric contact lenses are still being finalized and therefore not ready for use on a large scale.

The challenge is open with obvious exchange benefits in all areas of research. To date, results of basic biological research are often separated from clinical practice and would then need to create and implement a project of multidisciplinary clinical-biological integration.

Interesting therapeutic prospects may also result from the ability to integrate multi-level interventions in the biological rehabilitation (in particular in the case of glaucomatous optic neuropathy) and parasurgical/surgical therapy. Day after day more and more knowledge is expanding about the operation of the retinal neuronal network and its process of pathological deterioration, with sure progress not only in technological, but also pharmacological, surgical and biological field.

In the light of these findings, it is desirable that future glaucoma treatment will be focused on a more repairing and regenerating approach of loss visual and cell function, instead of one limited only to the mere IOP control.

## Author contributions

Both RN and FT gave their substantial contribution to conception and design of the manuscript and to the acquisition and interpretation of data and materials. Both authors gave their contribution in drafting the manuscript and in its critical revision for important intellectual content. All authors have approved the manuscript in its present form for publication. All authors agree to be accountable for all aspects of the work in ensuring that questions related to the accuracy or integrity of any part of the work are appropriately investigated and resolved.

### Conflict of interest statement

The authors declare that the research was conducted in the absence of any commercial or financial relationships that could be construed as a potential conflict of interest.
